# The Janus Face of Death Receptor Signaling during Tumor Immunoediting

**DOI:** 10.3389/fimmu.2016.00446

**Published:** 2016-10-31

**Authors:** Eimear O’ Reilly, Andrea Tirincsi, Susan E. Logue, Eva Szegezdi

**Affiliations:** ^1^Apoptosis Research Center, School of Natural Sciences, National University of Ireland, Galway, Ireland

**Keywords:** TNF-related apoptosis-inducing ligand (TRAIL), FAS (CD95), apoptosis, necroptosis, pro-survival signaling, immune surveillance, cancer

## Abstract

Cancer immune surveillance is essential for the inhibition of carcinogenesis. Malignantly transformed cells can be recognized by both the innate and adaptive immune systems through different mechanisms. Immune effector cells induce extrinsic cell death in the identified tumor cells by expressing death ligand cytokines of the tumor necrosis factor ligand family. However, some tumor cells can escape immune elimination and progress. Acquisition of resistance to the death ligand-induced apoptotic pathway can be obtained through cleavage of effector cell expressed death ligands into a poorly active form, mutations or silencing of the death receptors, or overexpression of decoy receptors and pro-survival proteins. Although the immune system is highly effective in the elimination of malignantly transformed cells, abnormal/dysfunctional death ligand signaling curbs its cytotoxicity. Moreover, DRs can also transmit pro-survival and pro-migratory signals. Consequently, dysfunctional death receptor-mediated apoptosis/necroptosis signaling does not only give a passive resistance against cell death but actively drives tumor cell motility, invasion, and contributes to consequent metastasis. This dual contribution of the death receptor signaling in both the early, elimination phase, and then in the late, escape phase of the tumor immunoediting process is discussed in this review. Death receptor agonists still hold potential for cancer therapy since they can execute the tumor-eliminating immune effector function even in the absence of activation of the immune system against the tumor. The opportunities and challenges of developing death receptor agonists into effective cancer therapeutics are also discussed.

## Introduction

The concept of immune surveillance was first proposed by Ehrlich in 1909 (1) and later refined by Burnet (2). They postulated that immune cells could both detect and eliminate malignantly transformed cells (2). During the past 20 years, analysis of both mouse models of cancer and primary human cancers have provided compelling evidence validating the immune surveillance concept. Studies have shown that immunocompromised mice lacking a functional interferon gamma (IFNγ) system (IFNγ receptor-1/alpha chain-deficient mice) or an intact T cell compartment are more susceptible to carcinogen-induced tumors, such as fibrosarcomas (3, 4). Furthermore, tumors formed in an immune-deficient background are more immunogenic than those formed in immunocompetent hosts, demonstrating that the immune system not only protects the host against tumor formation but also edits the arising tumor’s immunogenic characteristics.

The ultimate goal of tumor immune surveillance is to identify and eliminate transformed cells. The main effector immune cell types exerting cytotoxicity against malignant cells are cytotoxic T cells (CTLs) and natural killer (NK) cells. Although their mechanisms of activation differ, both CTLs and NK cells induce tumor cell death through death ligand cytokines of the tumor necrosis factor (TNF) ligand family (5, 6). However, while the immune system can potently limit early tumor formation, some tumor cells can escape by gaining resistance to death ligand-induced cell death. In such instances, the immune system-mediated presentation of death ligands can paradoxically promote tumor progression.

## The Role of Death Ligands in Tumor Immune Elimination

Tumor cell surveillance is facilitated by components of both the innate and the adaptive immune system, with the two systems having a complementary role in the recognition of transformed cells. While adaptive immune cells recognize specific antigens presented by class I MHC molecules on the surface of tumor cells (7), innate immune cells screen for more general markers of transformation, such as loss of MHC I expression, senescence, or cellular stress (8–10). The identified malignant cells are subsequently eliminated by immune effector cells; predominantly CD8^+^ CTLs and NK cells.

### Tumor Cell Recognition by NK Cells

NK cells are derived from the common lymphoid progenitor cell (11, 12) and are essential components of the innate immune system. As effectors of the innate immune system, NK cells recognize tumor cells *via* generic/ubiquitous stress markers through an array of antigen receptors (13). These antigen receptors are divided into two classes based on their effect on NK cell function: (1) *inhibitory receptors*, where ligand binding blocks NK cell activation and (2) *activating receptors*, which trigger NK cell activation and target cell killing following ligation (14).

Tumor cell identification by NK cells follows two major models, namely the “missing-self” and the “induced-self” models. The missing-self mechanism is based on the lack of self MHC molecules (MHC class I) on the surface of tumor cells (15). NK cells tolerate healthy tissues through recognition of self-MHC molecules (16) that activate inhibitory NK cell receptors, including killer cell immunoglobulin (Ig)-like receptors (KIRs) (17) and C-type lectin receptors (e.g., NKG2A/CD94) (18). Similar to virus-infected cells, tumor cells downregulate MHC I expression to escape recognition by the adaptive immune system (19). This, however, relieves KIR-mediated NK cell inhibition, permitting NK cell activation against the tumor cell.

Besides downregulation of MHC I molecules, tumor cells can also present “induced-self” antigens including MHC class I-related sequence A (MICA) and MICB (20). Tumor cell antigen upregulation can be triggered by cellular stresses including replicative stress (21) and endoplasmic reticulum stress (22). Induced self-antigens act as activating NK receptor ligands. For example, MICA and MICB are recognized by the NK cell activating receptor NKG2D, triggering cytotoxicity against the NKG2D ligand-carrying tumor cell (23). Additional activating receptors presented on NK cells include CD16 (Fcγ receptor III), which recognizes antibody-opsonized cells by binding to the Fc-region of the antibody triggering antibody-dependent cellular cytotoxicity (ADCC) (24). The CD56^dim^CD16^+^ NK cell subpopulation accounts for nearly 90% of all circulating NK cells in the peripheral blood (24, 25) and are regarded as the most cytotoxic subset. The high cytotoxic potential of the CD56^dim^CD16^+^ NK cells compared to the CD56^bright^ NK cell population is believed to be due to their high perforin and granzyme content (25).

### Tumor Recognition by Cytotoxic T Cells

The second effector cell type in tumor immune surveillance is CTLs. T cells, similar to NK cells, are derived from the common lymphoid progenitor cell (11), but form part of the adaptive immune response. As such, each T cell carries a single and unique antigen-recognizing T cell receptor (TCR) (26). When a T cell recognizes a specific tumor-associated antigen (TAA), it becomes activated and differentiates into an effector T cell, such as CD8^+^ CTL or type 1 and type 2 helper T cells (Th1 and Th2) (27). CTLs directly kill target cells following their TCR binding to the antigen displayed by MHC I molecules present on the tumor cell surface (28). CD4^+^ helper T cells have also been reported to kill target tumor cells, either directly, if the latter expresses MHC II (as the CD4 molecule can only recognize MHC II, not MHC I), or indirectly, when the tumor cell does not express MHC II. MHC II expression in tumor cells may be induced by cytokines, such as IFNγ. This direct effector function is the same as that used by CD8^+^ CTLs. The majority of tumor cell types are however expected to be MHC II negative. CD4^+^ T cells have been reported to also kill MHC II negative tumor cells *via* indirect activation of tumor-residing macrophages and NK cells (29). Aside from cell killing, the important function of CD4^+^ helper T cells is activation of CD8^+^ CTLs through secretion of cytokines (30, 31).

**Figure 1:**
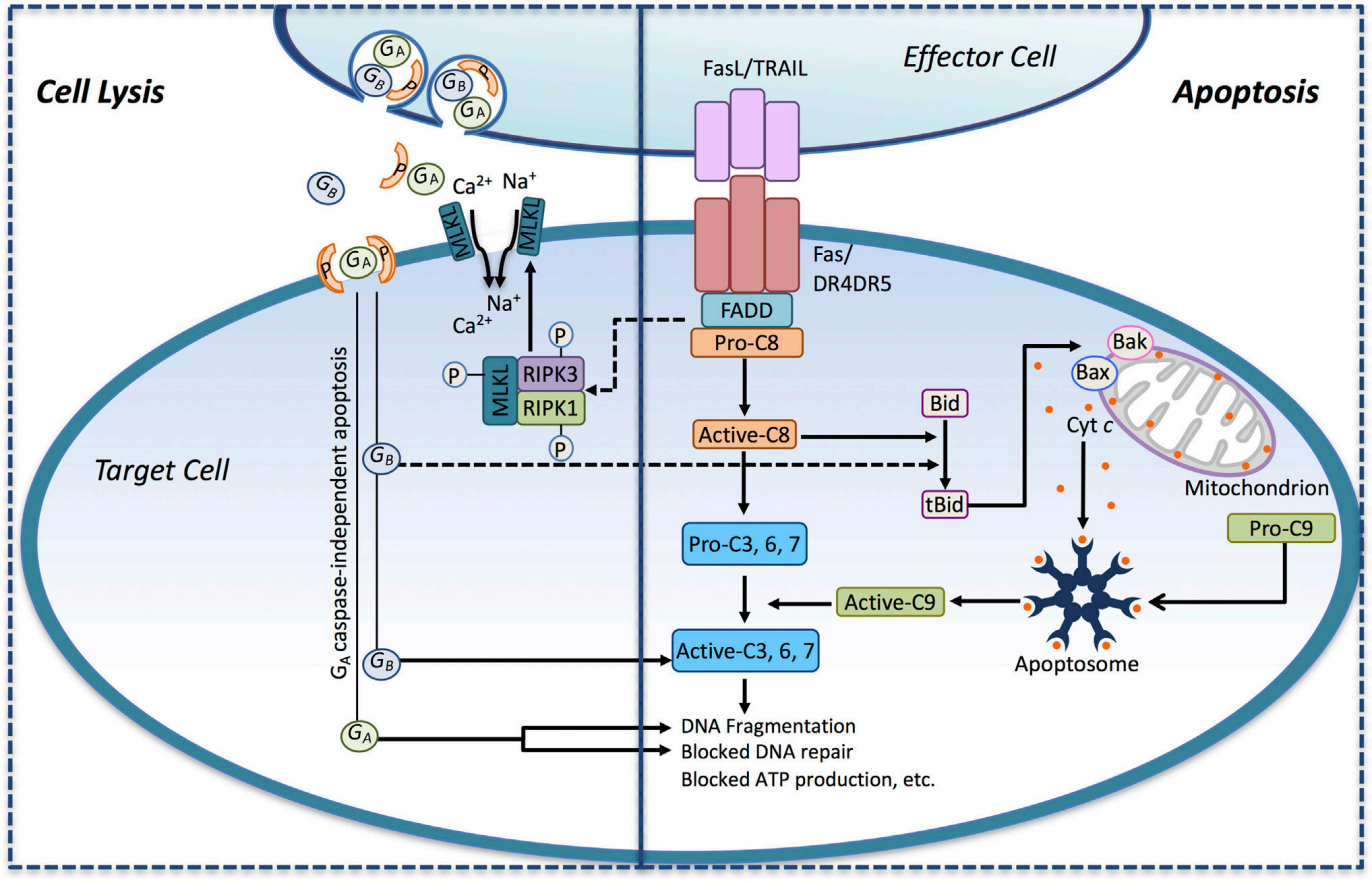
**Immune effector cells induce tumor cell death through apoptosis and necrotic-like cell lysis**. Death ligands (FasL, TRAIL) presented by immune effector cell interact with their corresponding death receptors (DRs) on the surface of the tumor cell and activate the extrinsic apoptotic pathway. Ligand binding induces DR activation leading to the recruitment of the adaptor protein FADD and pro-caspase-8. Pro-caspase-8 is converted to its active form (active-C8), and it cleaves the effector caspase-3, -6, and -7 to their active forms, thus engaging the executioner caspase cascade. Active-C8 can also trigger the intrinsic apoptotic pathway through the conversion of the BH3-only protein Bid to its active form, tBid. tBid, in turn, induces the formation of Bax/Bak megachannels in the outer mitochondrial membrane-releasing cytochrome *c* (Cyt *c*) into the cytosol. In conjunction with Apaf-1, pro-caspase-9 and Cyt *c* assembles into the apoptosome, where pro-caspase-9 becomes activated (active-C9) and released. Active-C9 assists active-C8 in the induction of the executioner caspase cascade. Activation of the DRs may also induce necrosis-like cell death through DR-mediated assembly of the necrosome complex consisting of RIPK1, RIPK3, and MLKL. In the necrosome, MLKL gets phosphorylated by RIPK1/RIPK3 leading to its oligomerization and translocation into the plasma membrane where it triggers Ca^2+^ and Na^+^ influx driving cell lysis. Recognition of the tumor cell may also trigger the secretion of perforin and granzymes from lytic granules toward the target cell. Secreted perforin forms pores in the target cell causing direct cell lysis and enabling the entry of the serine proteases granzyme A and B (G_A_ and G_B_) into the target cell. G_B_ can induce apoptosis by activating caspases through cleavage. G_B_ can also cleave Bid to tBid, thus engaging the mitochondrial apoptotic pathway. G_A_ can induce cell death in a caspase-independent manner by inducing DNA fragmentation and blocking DNA repair.

Regardless of the mechanism of NK/CTL activation or the tumor-specific antigen recognized, tumor cell killing occurs through two major pathways: (1) by perforin and granzyme-containing lytic granules or (2) *via* death ligand cytokines of the TNF superfamily (Figure 1).

#### Mechanism of Death Ligand-Induced Tumor Cell Death

Unstimulated NK cells can kill tumor cells by secreting the content of premade lytic granules. In response to tumor antigens and cytokines secreted by certain NK cell populations [CD56^bright^ NK cells (25, 32, 33)] and Th1 helper cells (34) in the tumor microenvironment, NK cells and CTLs also induce TNF death ligands to eradicate tumor cells (5, 6). These ligands, namely TNF, Fas ligand (FasL), and TNF-related apoptosis-inducing ligand (TRAIL) (35) activate their corresponding receptors present on the tumor cells, inducing apoptotic or necroptotic cell death (36).

##### Death Ligand-Induced Apoptosis

Death receptors (DRs), namely TNFR1, FAS, and DR4/5, belong to the TNF receptor superfamily of plasma membrane receptors. These receptors are generally characterized by a cytoplasmic sequence of approximately 80 amino acids known as the death domain (DD) (37). Signaling *via* TNFR1 is predominantly pro-survival linked to NF-κB signaling (38). It is the FasL receptor, FAS, and the two TRAIL receptors, DR4 and DR5 that primarily signal for cell death (37). The mechanism of cell death induced by FAS, DR4, and DR5 follows a highly conserved signal transduction pathway; the extrinsic apoptotic pathway.

In the absence of their ligand, DRs are present as monomers or preassembled dimers or trimers on the cell surface (39–41). Binding of the death ligand stabilizes the DR in trimeric or oligomeric complexes and induces a conformational change leading to DR activation. The activated receptor complex recruits the adaptor protein FADD and initiator caspases, caspase-8 and/or -10 leading to the formation of the death-inducing signaling complex (DISC), the activation platform for pro-caspase-8 (41).

FADD-mediated recruitment enables the dimerization of pro-caspase-8 driving its activation. Specifically, an intramolecular cleavage within the FADD-bound caspase-8 dimer liberates the small (p12) caspase homology domain, which is subsequently processed to the p10 catalytic subunit. The remaining 41/43 kDa caspase-8 intermediates present in the dimer then cleave one another in a trans-catalytic manner after the second DED releasing the p18 catalytic subunit. The two p18 units then associate with the two p10 subunits to form the heterotetrameric active caspase-8 complex (42).

Depending on the level of caspase-8 activation, cell death can be triggered either directly *via* effector caspase activation (referred to as type 1 mechanism) or indirectly through engagement of the intrinsic, mitochondrial-mediated pathway (referred to as the type II mechanism) (43). In the type II mechanism, the mitochondrial apoptotic pathway is activated by caspase-8-mediated cleavage of the BH3-only protein, Bid, producing truncated Bid (tBid) (44). tBid translocates to the mitochondria where it triggers oligomerization and activation of the Bcl-2 family members, Bax and Bak (45). The Bax and Bak oligomeres form pores in the outer mitochondrial membrane (Bax/Bak megachannels) through which cytochrome *c* is released into the cytosol (46). Cytochrome *c* together with dATP binds to apoptotic protease-activating factor-1 (APAF-1) inducing its oligomerization and formation of the pro-caspase-9 activating protein complex, the apoptosome (47). Active caspase-9 cooperates with DR-activated caspase-8/-10 in the activation of effector caspases and execution of cell death (48) (Figure 1).

##### Death Ligand-Induced Necroptosis

**Figure 2:**
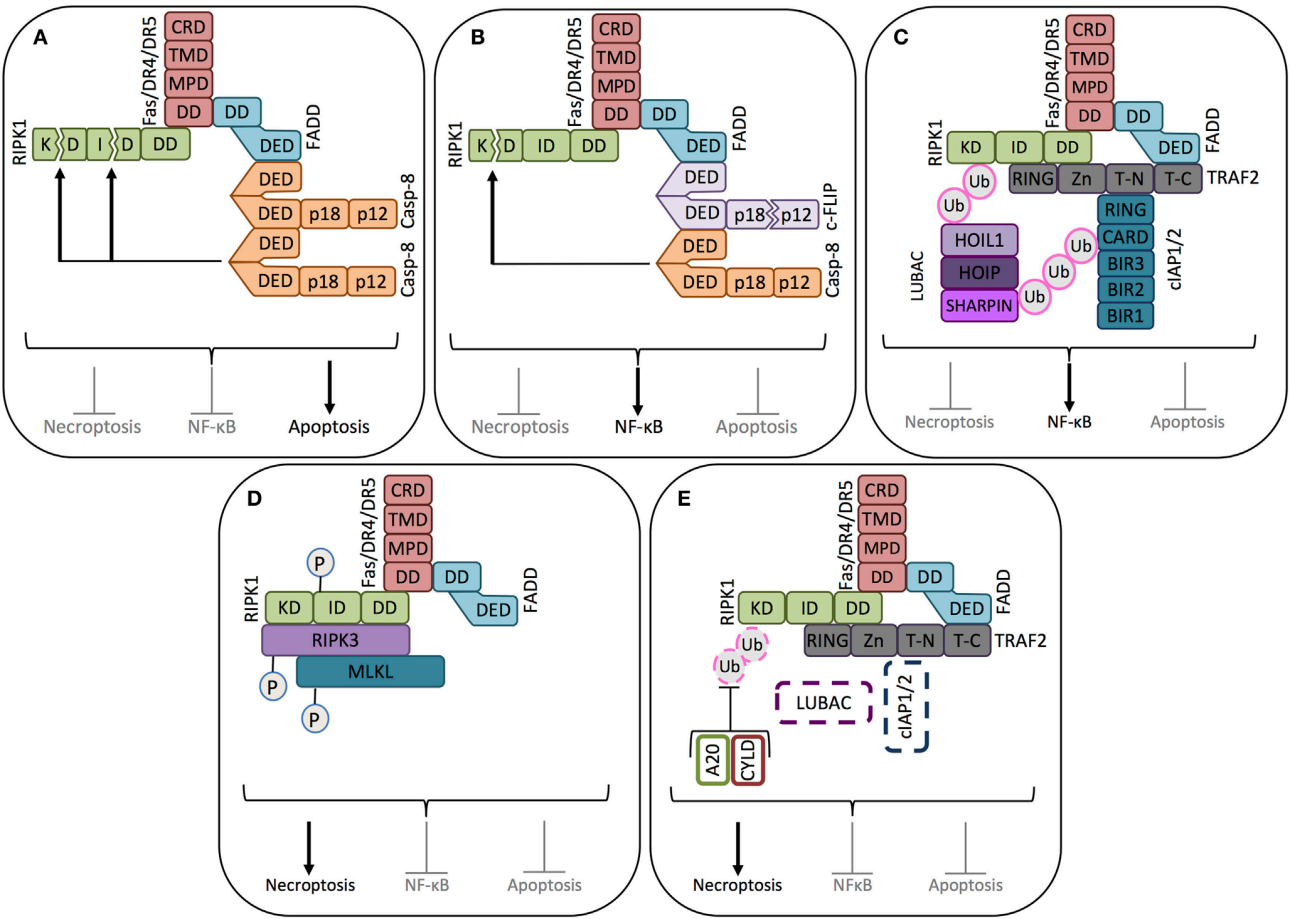
**Key signaling modes of death receptors**. **(A)** Apoptosis signaling mode. When pro-caspase-8 is abundantly expressed, binding of the death ligand (FasL or TRAIL, not shown) to its corresponding receptor induces the death domain (DD) of the receptor to complex with the DD of the adaptor protein FADD, allowing the recruitment, dimerization/oligomerization, and consequent activation of pro-caspase-8 (casp-8). Active caspase-8 inhibits RIPK1-mediated necroptosis and NF-κB activation by cleaving its kinase domain (KD) and intermediate domain (ID), driving apoptosis signaling. **(B)** NF-κB signaling mode-1. When cFLIP_L_ is highly expressed, together with pro-caspase-8, cFLIP_L_ is also recruited to the ligand-activated death receptor. Caspase-8 cleaves the small caspase homology domain of cFLIP_L_ (p12), generating cFLIP_p43_. cFLIP_p43_ and full-length caspase-8 form heterodimers that exhibit receptor-restricted and limited casapse-8 activity, not able to trigger the caspase cascade and apoptosis. The cFLIP_p43_-caspase-8 complex cleaves the kinase domain of RIPK1, but not the intermediate domain, thus blocking RIPK1-mediated necroptosis and driving NF-κB activity. **(C)** NF-κB signaling mode-2. In the absence of caspase-8, the adaptor protein TRAF2 recruits the ubiquitin ligases cIAP1/2. cIAP1/2 polyubiquitinates the proteins in the receptor complex to which the linear ubiquitin chain assembly complex (LUBAC) will bind. LUBAC and cIAP1/2 polyubiquitinate RIPK1, thus creating the platform for the assembly of the NF-κB activating protein complex. **(D)** Necroptosis signaling mode-1. In the absence of caspase-8 and cIAP1/2, RIPK1 recruited to the death receptor escapes caspase-8-mediated cleavage. In the absence of cIAP1/2, RIPK1 does not get ubiquitinated, which enables it to associate with RIPK3. After sequential trans- and autophosphorylation of RIPK1 and RIPK3, MLKL can be recruited to RIPK1 and RIPK3 to form the necrosome, thus triggering necroptosis. **(E)** Necroptosis signaling mode-2. In the absence of caspase-8, even in the presence of cIAP1/2, necroptosis can be induced if the cIAP1/2-conjugated polyubiquitin chains are removed from RIPK1 by the deubiquitinating enzymes A20 and CYLD. Once RIPK1 is deubiquitinated, it will induce the formation of the necrosome, as in **(D)**.

Besides activation of caspase-mediated apoptosis, DRs can also instigate plasma membrane permeabilization and a necrotic-like cell death (36). The necrosome, a cytosolic protein complex derived from the membrane bound DR complex, induces DR-dependent necrosis, or necroptosis. Core components of the necrosome include receptor interacting protein kinase 1 (RIPK1), receptor interacting protein kinase 3 (RIPK3), and mixed lineage kinase domain-like protein (MLKL) (49). Upon complex formation, RIPK3 phosphorylates MLKL leading to its oligomerization (50). Once phosphorylated, the 4-helical bundle domain (4HBD) of MLKL binds to negatively charged phosphatidyl inositol phosphates (PIP), anchoring MLKL to the plasma membrane where it drives cell lysis by triggering Na^+^ and Ca^2+^ influx into the cell (51). Ion influx can also occur through cation channels formed by MLKL itself, which are permeable to Na^+^, K^+^, and Mg^2+^ (52) and, *via* activation, of the plasma membrane cation channel, transient receptor potential cation channel subfamily M, member 7 (TRPM7) resulting in Ca^2+^ influx (53) (Figures 1 and 2D,E).

It should be noted that the preferential form of cell death induced by DRs is apoptosis, and the formation of the necrosome is only permitted in scenarios where caspase-8 is either not expressed (49) or its activation in the DISC fails (54). When active, caspase-8 cleaves RIPK1 within its kinase domains (KDs) and intermediate domains (IDs), thus blocking its function (55) (Figure 2).

#### Perforin–Granzyme-Mediated Cell Lysis

The death ligand and the granule-dependent cell lysis pathways work in a partially redundant, partially complementary fashion. Depending on the tissue, the effector immune cell type and cytokine/chemokine milieu, the relative contribution of the two pathways varies. Nonetheless, similar to the death ligand-induced cytotoxicity, lytic granules can also trigger both apoptotic and necrotic-like cell death.

Lytic granules contain perforin, a membrane pore-forming protein and a group of serine proteases, called granzymes (56). Recognition of the tumor cell by effector cells triggers the secretion of the content of the lytic granules (56). Perforin release enables pore formation in the target cell membrane facilitating either direct cell lysis or delivery of granzymes into the target cell’s cytosol (56). Granzyme A (GZMA) and Granzyme B (GZMB) are the most abundant subtypes present in NK cell and CTL lytic granules (56). GZMB can activate apoptotic cell death directly through processing of caspases (caspases-3, -6, -7, -8, -9, and -10) (56) or indirectly *via* cleavage of the BH3-only protein Bid and engagement of the mitochondrial apoptotic pathway (57, 58).

Unlike GZMB, GZMA induces target cell death in a caspase-independent manner by induction of DNA fragmentation and inhibition of DNA repair. GZMA does so by cleaving SET (SET nuclear proto-oncogene), an inhibitor of the DNase NME1 (expressed in non-metastatic cell 1/NM23-H1) leading to single-strand DNA breaks (59) (Figure 1). GZMA also targets the base excision repair enzyme apurinic endonuclease 1 (APE1) (60), poly- (ADP ribose) polymerase-1 (PARP-1) (61), as well as lamin A, B, and C (62), thus disabling DNA repair and disrupting the nuclear envelope culminating in cell death.

## Death Receptor Signaling in Tumor Immune Escape

While the immune system actively recognizes and eliminates most arising tumor cells (elimination phase of tumor immunoediting), some cells escape elimination and survive (63). The immune system can repress the growth of these escaped cells by creating a restrictive microenvironment where the tumor cells persists as dormant cells or continue to slowly evolve modulating their immunogenicity and other properties (equilibrium phase). The tumor immune suppression may however break or become exhausted, allowing unrestricted growth of the tumor cells (escape phase) (64, 65).

Of the death ligands, TRAIL has a distinct role in immune-mediated tumor elimination. In contrast to TNF and FasL, which can also induce death of healthy cells, TRAIL shows high specificity against malignantly transformed cells (66, 67). Malignant transformation has been demonstrated to selectively induce TRAIL sensitivity, in a cell-autonomous manner, driven by intracellular changes rather than cell-extrinsic factors, such as cytokines secreted by immune cells (e.g., IFNγ).

*In vitro*, experimental models of TRAIL induction during malignant transformation have shown TRAIL sensitivity can develop either during the immortalization stage or subsequently at the stage of oncogenic transformation induced by RAS (68, 69). Similarly, the transformation from premalignant colorectal adenoma cells to colorectal carcinoma cells was accompanied by an increase in TRAIL sensitivity (70). How TRAIL sensitivity increases during malignant transformation is not fully understood. Activation of the mitogen-activated protein kinase (MEK) (69), decreased expression of antiapoptotic proteins *via* inhibition of eukaryotic elongation factor (eEF2) (71), or inhibition of protein phosphatase 2A activity (PP2A) (72) have all been implicated as possible mediators. While the evidence for the involvement of these processes, especially PP2A, is compelling, the signaling pathways are not yet fully elucidated with further studies required to identify key components.

Importantly, there is substantial evidence indicating that transformation-driven TRAIL sensitivity is a key contributor to tumor immune elimination. For example, TRAIL neutralization or deficiency promoted tumor development in a mouse model of carcinogen methylcholanthrene (MCA)-induced fibrosarcoma (73). Because immunoediting eliminates evolving tumor cells *via* TRAIL-mediated killing (74), this selective pressure can result in tumor cells acquiring resistance to death ligand-mediated cytotoxicity.

### Counteracting Death Ligand-Induced Apoptosis

Tumor cells are exposed to a broad range of cellular stresses triggered as a result of oncogenic transformation, genetic instability or nutrient deprivation. To survive under such stress conditions, tumor cells induce the expression of antiapoptotic proteins. For example, a large proportion of tumors display increased expression of antiapoptotic Bcl-2 proteins (75) or the caspase-3 inhibitory protein, XIAP (76). These proteins confer resistance to various death stimuli. Beyond these general antiapoptotic mechanisms blocking apoptosis signaling at the stage where they converge, tumor cells develop mechanisms that provide specific protection against death ligand-mediated immune attack by (1) reducing the expression of DRs or essential death mediators, most notably caspase-8, (2) by impaired function of DRs, or (3) by blocking the transmission of the death signal from the DR. Examples of each of these processes are discussed below.

#### Loss of Essential Death Mediators

Tumor cells can prevent DR activation by inducing cleavage of death ligands from the surface of the immune effector cells. FasL and TRAIL can be cleaved off the surface of immune cells by matrix metalloproteinases (MMP)-3, -7, and -9 as well as ADAM-10 and 17 (a disintergrin and MMP) (77–79). While retaining their trimeric structure, metalloprotease-cleaved, soluble TRAIL and FasL have a 100- to 1000-fold lower cytotoxic potential in comparison to their membrane-bound counterparts (80). Furthermore, soluble FasL may even block the cytotoxic effect of membrane-bound FasL by competing with it for receptor binding (81) or directly promoting tumor progression by inducing pro-survival signal transduction upon receptor binding (for further details, please see “Death Receptor Signaling Driving Tumor Growth and Progression”) (82).

Death ligand-mediated cytotoxicity can also be controlled in tumor cells through regulation of DR expression. Indeed, reduced FAS expression has been reported in colon carcinomas (83) and in cutaneous T-cell lymphoma (CTCL) (84). Loss of TRAIL receptor expression on the other hand is not a common occurrence in cancers, and they may even be overexpressed in some cases (85). The examples of reduced TRAIL receptor include loss of DR4 expression in lung squamous cell carcinoma (86) and reduced DR4 and DR5 expression in hepatocellular carcinoma (HCC), which correlated with shorter overall 5-year survival of patients (87). Reduced FAS, DR4, and DR5 expression is typically caused by gene and/or promoter CpG methylation or trimethylation of histone 3 on lysine 9 (H3K9me3), while gene deletion was rarely detected (83, 86, 88). Activating RAS mutations have also been associated with silencing of the FAS gene by driving methylation of the FAS promoter region (89). A genome-wide RNA interference (RNAi) screen in KRAS-transformed NIH3T3 cells identified 28 genes driving KRAS-mediated epigenetic silencing of FAS; many of them are well-known cancer-associated epigenetic regulators, such as DOT1-like histone H3K79 methyltransferase (DOT1L), enhancer of zeste 2 polycomb repressive complex 2 subunit (Ezh2), sirtuin 6 (Sirt6), DNA methyltransferase 1 (DNMT1), and nucleophosmin 2 (NPM2). All 28 genes appeared to be essential for FAS gene silencing as knockdown of any one was sufficient to restore FAS expression (90).

Regulation of DR expression is not the only mechanism employed by tumors to evade death ligand-induced cell death. Gene deletion or silencing, *via* CpG methylation, of caspase-8 has also been reported. For example, caspase-8 promoter methylation is well described in neuroblastoma (91) and MYCN-driven childhood medulloblastoma (92). Loss of caspase-8 expression was also found in small cell lung carcinoma (SCLC), but interestingly, was completely absent in non-SCLCs (93). In line with these findings, MYCN amplification has been reported in SCLC (94, 95), while it is rarely occurring in NSCLC (96). It should be noted, however, that the association between caspase-8 gene silencing and MYCN status of SCLC has not been established.

#### Impaired Death Receptor Function

Manipulation of DR function is probably the most frequent mechanism employed to escape death ligand-mediated immune elimination. While FAS signaling is largely controlled through its expression, the function of DR4 and DR5 is predominantly controlled posttranslationally. Ligand binding and the consequent activation of DR4 and DR5 is kept in check by two membrane-bound decoy receptors, DcR1 and DcR2. These decoy receptors lack a functional DD and, therefore, are unable to induce apoptosis. The actions of DcR1 and DcR2 are twofold. First, by sequestering TRAIL, they prevent DR4/DR5 activation (97) and second, through formation of heteromeric complexes with the death-inducing receptors, they impair DR complex confirmation upon ligand binding (98).

DcR1 and DcR2 overexpression has been reported in several tumor types, including acute promyelocytic leukemia (APL) (99) and prostate cancer (100). Ubiquitous DcR1 (but not DcR2) expression has also been reported in the tumor-surrounding stroma of various cancers including breast, liver, pancreatic, ovarian, and prostrate (101). While deficient mTRAIL-R expression in mice promotes metastasis (102), it is not known if DcR1 and/or DcR2 can lend similar properties to tumors. Nonetheless, the fact that there are multiple decoy receptors for a single death ligand implies that tight regulation of this pathway is vital.

Osteoprotegerin (OPG), a soluble decoy receptor for receptor activator of nuclear factor-κB ligand (RANKL), can also bind TRAIL, although with a lower affinity (103, 104). While OPG’s main biological function is to control osteoclast maturation/activity, it has also been implicated in breast cancer progression. Breast cancer cells have been reported to secrete OPG, which, through sequesterization of TRAIL, furnished the breast cancer cells with more aggressive growth and metastatic potential (104).

The activity of FAS can also be controlled by a decoy receptor, DcR3. Similar to OPG, DcR3 is a secreted protein and a decoy receptor for multiple TNF family cytokines including vascular endothelial growth inhibitor (VEGI/TL1A/TNFSF15), FasL, and LIGHT (TNFSF14). DcR3 overexpression has been reported in many cancers including lung and colon cancers, Epstein–Barr virus (EBV) or human T-cell lymphotropic virus-1 (HTLV-1)-associated lymphomas, gliomas, and pancreatic adenocarcinomas as well as bone and soft tissue sarcomas (105). There is compelling evidence indicating that DcR3 overexpression enables the formation of distant metastases and associates with reduced overall survival in cancer patients (105).

It should be noted that the effect of the soluble and ligand-promiscuous decoy receptors, OPG and DcR3, on tumor metastasis, is more complex than sequestration of TRAIL and FasL. In the case of OPG, bone resorption is a key contributor of bone metastasis, and OPG acts as an inhibitor of this process (106). Thus, OPG may be required to provide TRAIL resistance to tumor cells disseminated from the primary tumor, but not for bone metastasis. The function of DcR3 is even more complex. DcR3 can induce angiogenesis and block T cell costimulation induced by TL1A (107). DcR3 also drives reverse signaling *via* binding to its membrane-bound ligands that can trigger dendritic cell (DC) death *via* cross-linking of heparan sulfate proteoglycan (HSPG) on DCs (108).

Mutations that impair apoptosis signaling by DRs are alternative mechanisms cancers employ to evade immune elimination. To date, over 180 mutations have been identified on the FAS gene and are predominantly clustered on the exons that code the DD (exons 8 and 9) (109). FAS mutations are rarely homozygous and are not exclusively restricted to cancer with several mutations reported in the autoimmune disease autoimmune lymphoproliferative syndrome (ALPS) (110). Mutations affecting the folding of the DD or its interaction with the DD of FADD act in a dominant negative manner, i.e., they are able to impair the functionality of the non-mutated receptors by forming heteromeric complexes with them (40). The presence of even a single defective protein in the oligomeric DR complex is sufficient to cripple the active receptor conformation or hinder the recruitment of downstream adaptors (FADD and caspase-8) in the correct stoichiometry for DISC formation (40).

Mutations in TRAIL receptors have also been detected in cancers, although not as numerous as for FAS. For example, DR5 mutations have been reported in NSCLC (111), non-Hodgkin’s lymphoma (112), breast cancers (113), and head and neck cancers (114). Similar to FAS, TRAIL receptor mutations (E355K, E367K, K415N, and L363F) are typically localized to the DD and may induce conformational changes including decreased protein backbone flexibility, decreased exposure of FADD’s DED for caspase-8 binding, and reduced binding affinity of DR5 DD binding to FADD (115), thus, significantly decreasing the ability of these cells to undergo TRAIL-induced apoptosis (116). Other studies have reported DR5 mutations to be rare in cancers. For example, mutations in DR5 are uncommon in HCC (117), and while the DR5 gene is frequently found in the loss of heterozygosity (LOH) region at 8p21–22 in bladder cancer, the DR5 gene itself was not mutated (118).

#### Targeting the Transmission of the Cell Death Signal

Cellular FLICE-inhibitory protein (cFLIP), a pseudo-caspase with a high homology to pro-caspase-8 (119), primarily functions to control DR-mediated caspase-8 activation. Increased expression of cFLIP is frequently observed in numerous cancer types, such as melanoma (120) and prostate cancer (121). cFLIP has three main splice variants, namely the 55 kDa cFLIP-long (cFLIP_L_), the 26 kDa cFLIP-short (cFLIP_S_), and the 24 kDa cFLIP-Raji (cFLIP_R_) (122). Similar to pro-caspase-8, all three cFLIP variants possess two N-terminal DEDs. In addition, cFLIP_L_ also contains large and small caspase homology domains (123), but due to inactivating mutations in the catalytic- and substrate-binding sites, possesses no enzymatic activity (124). All cFLIP splice variants compete with caspase-8 for binding to FADD through their DEDs (125) and can also form heteromeres with caspase-8 within the DISC. In the case of FLIP_S/R_, heterodimerization fails to activate pro-caspase-8 as the conformational change in the caspase catalytic domain of pro-caspase-8 cannot take place. Thus, FLIP_S/R_ acts as dominant negative inhibitors (42).

The function of cFLIP_L_ in regulating pro-caspase-8 activation is more intricate. Within the cFLIP_L_-pro-caspase-8 heterodimer, the caspase homology domain of cFLIP_L_ induces a conformational change within procaspase-8 enabling partial enzymatic activity (42) resulting in c-FLIP_L_ cleavage to an N-terminal 43 kDa fragment (p43) and a C-terminal p12 fragment (126). The activation process halts at this point, as cFLIP_L_ cannot implement the same cleavage on pro-caspase-8. Thus, the cFLIP_(p43)_-caspase-8 heterodimer remains tethered to the DISC and is unable to activate the apoptotic caspase cascade (42). Instead, the FLIP_(p43)_:caspase-8 heterodimer is an effective inducer of NF-κB activation (126) (Figure 2B). While fully processed caspase-8 can inhibit all RIPK1 functions, by cleaving it in its KD and ID, the FLIP_(p43)_:caspase-8 heterodimer only cleaves RIPK1 in the KD domain. This cleavage, while still permitting recruitment of RIPK1 to the DR and downstream NF-κB activation (55), blocks RIPK1-driven necroptosis (127) (Figure 2B).

Inhibitors of apoptosis proteins (IAPs) are defined by the presence of a baculovirus IAP repeat (BIR) domain. The primary mammalian IAPs are cellular inhibitor of apoptosis 1 (cIAP1, BIRC2), cIAP2 (BIRC3), X-linked IAP (XIAP, BIRC4), and survivin (BIRC5). cIAP1, 2, and XIAP contain three BIR domains while survivin has one (128, 129). Initially, IAPs were classified as caspase binding and inhibitory proteins. However, it is now understood that XIAP is the only member of the family to function in such a manner. Through BIR domain interactions, XIAP can bind the initiator caspase, caspase-9, and the effector caspases, caspase-3 and -7, preventing their downstream signaling (130). Although cIAP1 and 2 can also bind caspases, their predominant function in DR signaling is polyubiquitination of protein substrates through their E3 ubiquitin ligase activity (131).

Following recruitment to the DISC, through the adapter protein TRAF2, cIAP1/2 ubiquitinate RIPK1 and other components of the complex. Components of the NF-κB activating machinery assemble on these ubiquitin chains leading to NF-κB activation (132). Once activated, NF-κB elevates expression of cFLIP, antiapoptotic Bcl-2 proteins, and XIAP providing apoptosis resistance to the tumor cell (133) (Figure 2C).

Recruitment of cIAP1/2 and the consequent ubiquitination of RIPK1 is a key switch between cell death and cell survival signaling. The first decision point in DR-induced signal transduction is the activation of pro-caspase-8. If caspase-8 can be recruited in homodimeric or homo-oligomeric complexes, i.e., not in cFLIP heteromeres, it will undergo full activation and inhibit RIPK1-mediated NF-κB activation (55).

In the absence of caspase-8 activation, the cell can either undergo death *via* necroptosis (134) or induce NF-κB activity and survival signaling (135) with the outcome dependent on the ubiquitination status of RIPK1 (Figure 2D). In the absence of cIAP1/2 or when RIPK1 is deubiquitinated by deubiquitinating enzymes, such as cylindromatosis (CYLD) or A20, RIPK1 can associate with RIPK3 and MLKL and induce necroptosis (136). Thus, high expression of cFLIP, cIAP1/2, or caspase-8 gene silencing in cancer cells will propel survival signaling upon DR activation (Figure 2E).

### Death Receptor Signaling Driving Tumor Growth and Progression

Recent findings have highlighted that DR-driven survival signaling does not only provide resistance against death ligand-mediated immune elimination but also equips the tumor cells with enhanced migratory and invasive potential. Induction of migration and invasion has been reported for both TRAIL and FasL.

**Figure 3:**
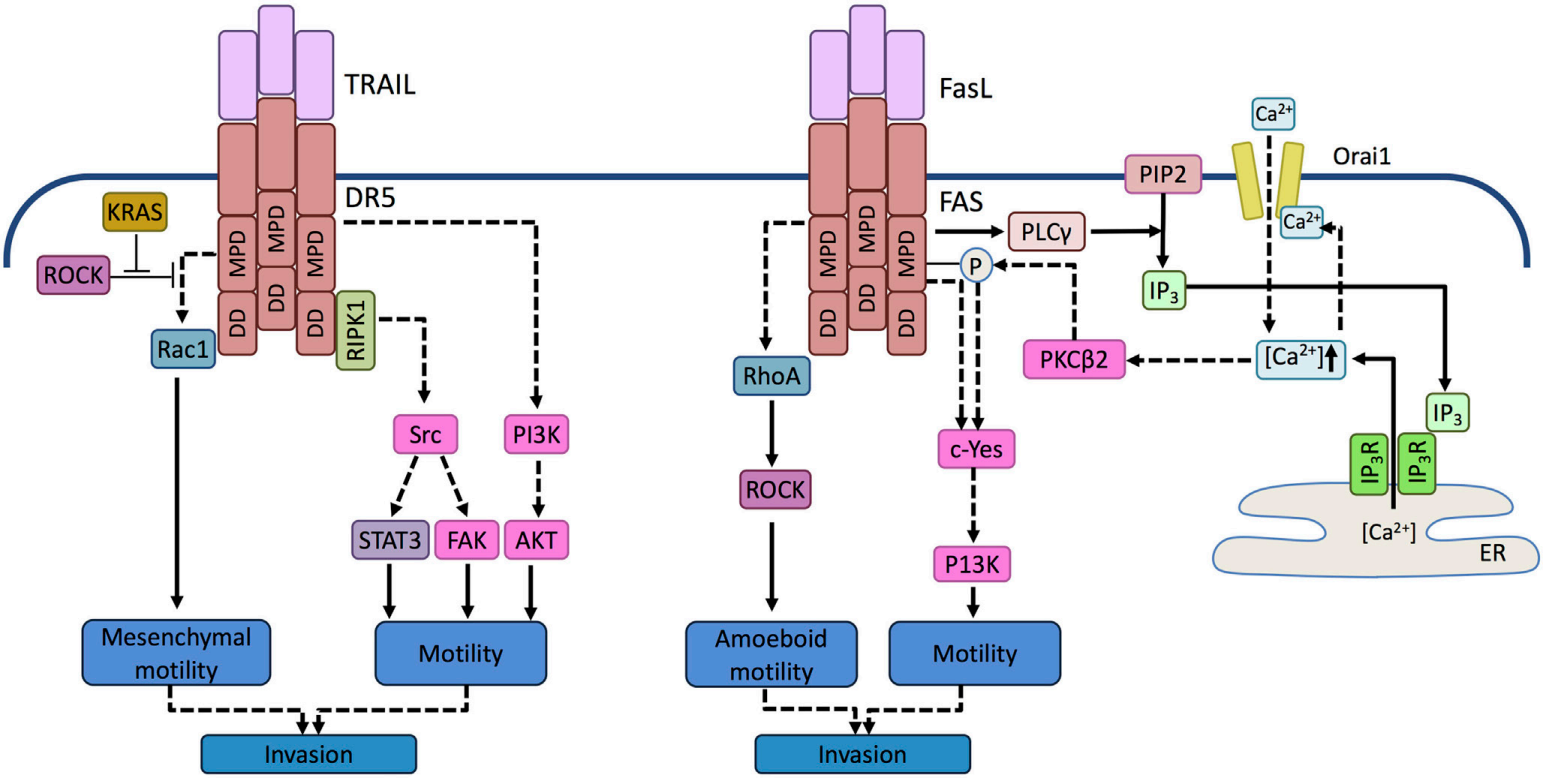
**Prometastatic signaling mediated by death receptors**. Activation of DR5 in TRAIL-resistant tumor cells can trigger activation of the tyrosine kinase Src in a RIPK1-dependent manner and phosphatidyl inositol 3 kinase (PI3K) in a RIPK1-independent manner. Activation of Scr induces signal transducer and activator of transcription 3 (STAT3) and focal adhesion kinase (FAK)-mediated motility, while PI3K promotes motility and invasive potential by activating Akt. In KRAS-driven tumor cells, KRAS-mediated inhibition of ROCK allows Rac-1 activation upon TRAIL receptor activation. Activation of Rac1 is mediated by the membrane proximal domain (MPD) of the TRAIL receptor independent of the death domain (DD). RAC-1 drives cell motility associated with mesenchymal morphology and invasive potential. Similar to TRAILR, in FasL-resistant cells, ligand binding by FAS can activate a Src family kinase, c-Yes. c-Yes activation is promoted by phospholipase C gamma (PLCγ) activated through the membrane proximal domain of FAS. PLCγ cleaves membrane phosphatidyl inositol bisphosphates (PIP2) into inositol trisphosphate (IP3). IP3 translocates to the endolasmic reticulum (ER) and opens the ER store calcium channel, IP3 receptor. The released calcium activates the plasma membrane Ca^2+^ channel Orai-1 causing a Ca^2+^ influx that induces a local Ca^2+^ signal in the vicinity of FAS and activates protein kinase C beta 2 (PKCβ2). PKCβ2 attenuates apoptosis signaling by phosphorylating FAS and triggers c-Yes activation that drives motility. FAS can also activate the small GTPase RhoA, but in contrast to DR5/TRAILR signaling events, it leads to the activation of ROCK and consequent amoeboid motility.

mTRAIL-R (MK/mDR5), the single murine TRAIL DR, has been shown to promote invasion and metastasis in KRAS^G12D^-driven non-SCLC (NSCLC) and pancreatic ductal adenocarcinoma (PDAC) (137). In KRAS mutant cells, upon activation, TRAIL receptors triggered the activation of RAS-related C3 botulinum substrate (Rac1), a small GTPase important for cytoskeletal rearrangement and cell motility. Rac1 is known to drive mesenchymal-type cell motility described by elongated cellular morphology and requirement of extracellular proteolysis, as opposed to amoeboid cell movement, which is associated with rounded cell morphology and lower dependency on proteases. Amoeboid cell motility, on the other hand, is driven by Rho kinase (ROCK) in a dominant manner, since ROCK can block mesenchymal motility by inhibiting Rac1 activity (138). Transformation by oncogenic KRAS has been found to lead to inhibition of ROCK (139), thus allowing TRAIL-induced Rac1 activation and mesenchymal-type cell motility (137) (Figure 3).

In addition to Rac1 activation, RIPK1-driven Src, STAT3, and focal adhesion kinase (FAK) activation as well as RIPK1-independent activation of phosphatidyl inositol 3 kinase, Akt and Erk have been shown to trigger DR5-dependent migration and matrigel invasion of NSCLC cells (140). Inhibition or knockdown of RIPK1, Src, STAT3, and PI3K either fully abolished or reduced TRAIL-driven cell migration. Interestingly, activation of these kinases and the consequent migratory behavior was only apparent in TRAIL-resistant cells. Furthermore, inhibition of Src did not sensitize cells to TRAIL-induced death indicating that the two DR-driven signaling pathways may be regulated independently (140).

It should be noted that the two TRAIL receptors, DR4 and DR5, may have a differential potential in inducing tumor cell migration. It appears that DR5, especially its membrane proximal domain (MPD), is the main driver of migration and DR4 does not transmit the same signal. In line with this, Spierings and colleagues reported that high DR5 expression was associated with an increased risk of death in NSCLC (141), although further research is necessary to substantiate these findings.

Similar properties of the FAS receptor have been reported. The “alternative” migratory signal has been reported in two main scenarios. First, if FAS is activated by soluble FasL following its shedding from the surface of immune effector cell in the tumor microenvironment (142). Second, when the DD of FAS contains a loss-of-function mutation. Similar to DR5, the membrane proximal domain (MPD) of the receptor is believed to be responsible for the pro-migratory signal transduction. Upon activation, the MPR recruits the Src kinase family member c-Yes resulting in PI3K pathway activation. This process can also trigger Ca^2+^ influx through the plasma membrane leading to the activating protein kinase C β2 (PKCβ2). PKCβ2, in turn, halts DISC formation by phosphorylating FAS (143, 144) (Figure 3).

Involvement of ROCK in FAS-mediated motility has also been proposed, although in the opposite manner, whereby FAS ligation was found to activate the small GTPase, RhoA driving ROCK activation. Active ROCK subsequently activated the Na^+^/H^+^ exchanger NHE1, thus driving cell motility (145).

While the ability of DRs to trigger tumor cell motility is well established, the composition of this non-canonical, “motility-inducing signaling complex (MISC)” is not. The effect of ROCK in DR-mediated motility, i.e., whether it is activation or inhibition of ROCK that drives it, as well as the involvement of caspase-8 in DR-mediated kinase activation is currently controversial (146).

## Targeting Death Receptor Signaling for Anticancer Therapy

**Figure 4:**
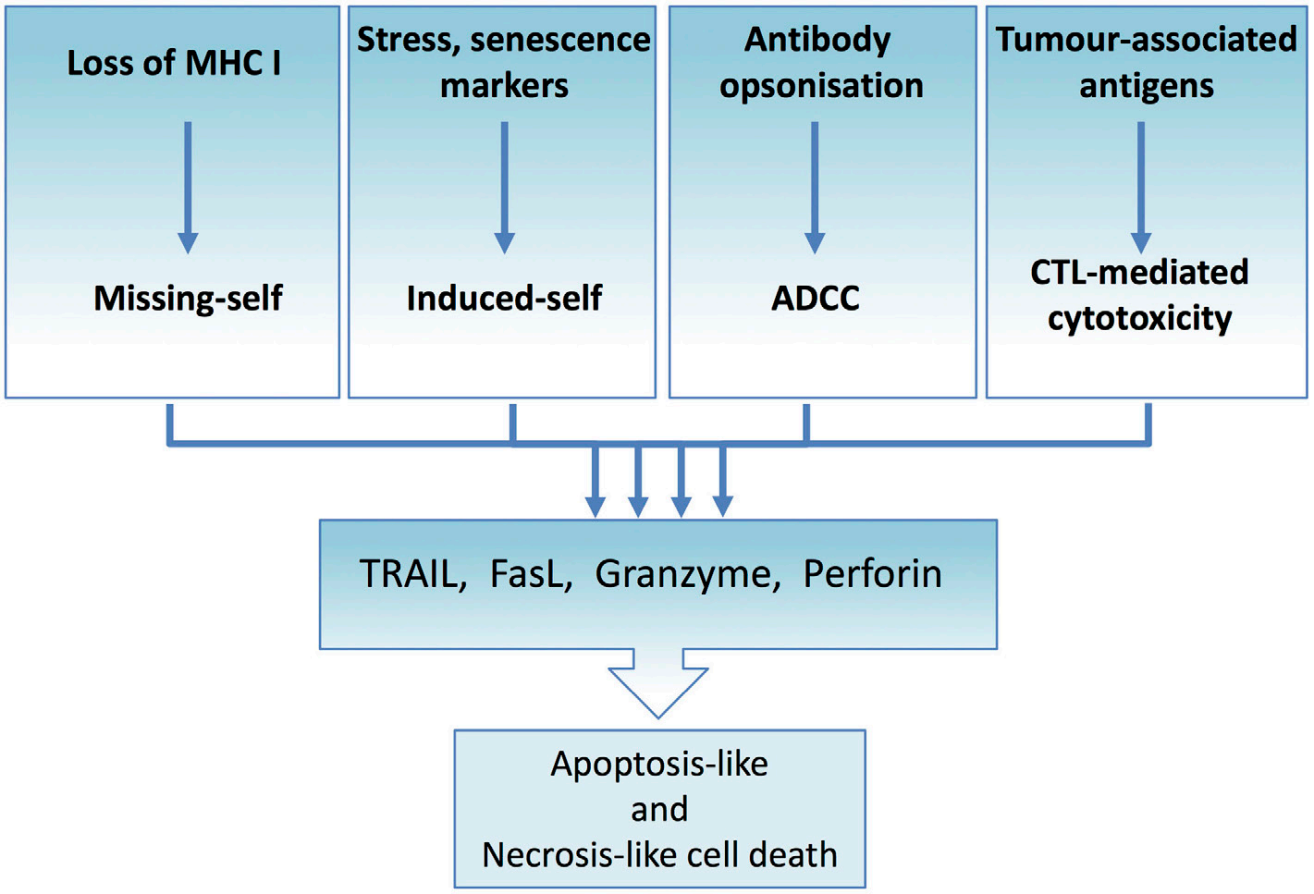
**Tumor immune elimination converges on death ligand and perforin/granzyme-mediated cytotoxicity**. All immune effector-mediated tumor elimination, regardless of the signal or the mechanism, converges and depends on the perforin and granzyme-induced cell lysis or death ligand-induced apoptosis or necroptosis. Tumor cells resistant to these cell death mechanisms escape tumor elimination. At the same time, effector cells can expose tumor cells to the full complement of cytotoxic molecules, thus maximizing the range of targetable tumor cells.

Priming and activation of the immune system against cancer cells hold great promise. The potential of the immune system to identify and kill individual tumor cells and retain the memory of TAAs, which enables the elimination of metastasized and reactivated tumor cells, is immense. Removal of malignant cells, regardless of the mechanism of recognition, depends on death ligands and/or lytic granules (Figure 4). Therapeutic replication of the cytotoxic immune effector functions would bypass the intricate and complex tumor immune interactions and execute tumor elimination without the need to prime the immune system against the tumor.

This proposal has instigated extensive research to develop DR agonist-based therapeutics with initial studies focusing on recombinant death ligands as evidenced by the number of clinical and preclinical studies (Table S1 in Supplementary Material). Although activation of TNFR1 and FAS with recombinant ligands and with agonistic antibodies against the receptor, such as Jo2, was highly efficient in killing tumor cells, they also induced systemic toxicity due to induction of severe inflammatory reactions by TNF and of extensive apoptosis in hepatocytes by FAS agonists (147, 148). The mechanism of FAS agonist-induced fulminant hepatitis is now understood in deeper detail revealing a multifold action including direct effect on hepatocytes and indirect, ADCC through the FcgammaRIIB receptor targeting the sinusoidal endothelial cells and causing hemorrhage (149). These findings led to the development of recombinant FasL and agonistic anti-Fas antibodies engineered for targeted delivery to the tumor cells (150). The third death ligand, TRAIL, on the other hand showed high specificity against tumor cells and minimal toxicity against normal tissues, including hepatocytes, in clinical trials. However, its excellent safety profile was not matched with its expected efficacy (151).

Thus, the strategy to directly induce immune effector cell-mediated tumor cell killing was revisited. Recent advances in our understanding of DR regulation and function have enabled refinement of these therapies. We now know DR agonists should mimic the receptor activation mechanism of the membrane-bound native ligand, avoid sequestration by decoy receptors, effectively target and accumulate in the tumor tissue, and ideally promote activation of the immune system against the tumor.

The importance of receptor selectivity has been recognized for some time and selective agonists against the TRAIL DRs either in form of agonistic antibodies or engineered ligand variants have been developed (101, 152). The ability of these agonists to fully replicate the receptor-activation mechanisms of the membrane-bound native ligand, however, has not been fully elucidated yet. Agonistic antibodies are believed to better mimic the function of membrane-bound TRAIL. Nonetheless, the early stage clinical trials with agonistic antibodies also met with limited efficacy, confirming that while receptor selectivity is important, specific tumor targeting is probably also essential (153).

Based on the observation that adoptive T-cell therapy often fails to induce meaningful anticancer responses in cancer patients, the Bremer laboratory developed fusion proteins of TRAIL where the extracellular portion of TRAIL was fused with either an antibody fragment recognizing the T cell surface molecule CD3 or with K12, the ligand of another T cell marker, CD7 (154). This fusion strategy enables TRAIL to bind to the surface of T cells and functionalize them against tumor cells. Xenograft mouse studies showed accumulation of the TRAIL-functionalized T cells at the tumor site and robust antitumor activity (154). Importantly, 80% of the mice treated with the ant-CD3-TRAIL-functionalized T cells survived over 70 days and the treatment had no noticeable toxicity.

To address selective targeting of TRAIL to tumor cells, new approaches utilizing bispecific antibodies are also emerging. For example, the development of a bispecific antibody against melanoma chondroitin sulfate proteoglycan (MCSP) and DR5 has recently been reported. MCSP is highly expressed on the surface of almost 90% of melanomas, but not on normal melanocytes (155, 156). MSCP × DR5 antibody bound selectively and with high affinity to MSCP^+^ melanoma cells where it exerted strong and selective DR5-dependent cytotoxic activity against MCSP-expressing melanoma cells. Furthermore, the antibody could also trigger NK-cell-mediated ADCC through recognition of its Fc-region by Fcγ-receptor expressing immune cells (NK cells). This approach offers a novel immunotherapeutic tool *via* coupling of three cooperating processes: delivering the DR agonist to the malignant cell population, potent activation of DR5-mediated cell death signaling, and recruitment of Fcγ-receptor-carrying immune cells that can mount an immune response against the tumor cells (for a summary of current formulations of DR-agonists, please see Table S1 in Supplementary Material).

The development of DR agonist-based cancer therapeutics is ongoing. A major challenge in DR agonist-based cancer therapeutics is to design therapies, which while promoting selective DR mediated cytotoxicity against tumor cells avoid unintentional triggering of pro-survival pathways. The ongoing focus on understanding how cell death versus pro-survival decisions are made downstream of DRs may enable the development of drug combinations that block the non-death signaling while inducing cell death.

The remarkable effect of the immune checkpoint inhibitors has also highlighted the potential of the immune system to eradicate tumors once the suitable conditions established offering the concept of combination therapies with DR agonists. There is also increasing evidence for the synergistic interaction between cell death pathways, for example between TRAIL and FasL (157), as well as the immune-activating potential of different cell death forms. Similar interaction may exist between the perforin–granzyme and death ligand cytotoxic pathways that future therapies might exploit.

There are still a number of open questions concerning DR signaling. While the signal transduction pathways mediated by TNFR1 are increasingly understood, the range and composition of non-apoptotic signal transducing protein complexes activated by TRAIL receptors and FAS are poorly elucidated. It is imperative that we have a thorough understanding of these complexes and the triggers promoting their formation to avoid accidental activation of pro-survival pathways when therapeutically targeting DRs. We also need to dissect the cytotoxicity mechanisms induced by different formulations of DR agonists, whether it is direct activation of the extrinsic apoptotic pathway, necroptosis, or ADCC induced by NK cells recognizing tumor cells opsonized by agonistic anti-DR antibodies. Overall, we need to understand the role of death ligands and DRs in the context of tumor immune interaction in order to develop DR agonists into an effective cancer therapeutic.

## Author Contributions

The concept, content, and structure were formulated by ES. ES, EOR, AT, and SL contributed to literature research, interpretation, writing, editing, and proofreading of the manuscript. EOR, AT, and ES generated the figures.

## Conflict of Interest Statement

The authors declare that the research was conducted in the absence of any commercial or financial relationships that could be construed as a potential conflict of interest. The reviewer SN and handling Editor declared their shared affiliation, and the handling Editor states that the process nevertheless met the standards of a fair and objective review.
