# Supplementary material for: The Janus Face of Death Receptor Signaling during Tumor Immunoediting
Source: Front Immunol. 2016 Oct 31;7:446. doi: 10.3389/fimmu.2016.00446 (PMC5086583; doi:10.3389/fimmu.2016.00446)
Supplement: Supplementary file 1 [file Table_1.DOCX]

**Supplementary Table 1: Death receptor agonist-based therapeutics and their clinical development**

**TRAIL-R1 (DR4) monoclonal antibodies**

| **Name** | **Cancer type tested** | **Combination/**  **monotherapy?** | **Major outcomes** | **Clinical Trial Phase** |
| --- | --- | --- | --- | --- |
| Mapatumumab (HGS1012/ HGS-ETR1) | Advanced solid tumours | Cisplatin, gemcitabine | In combination with cisplatin/ gemcitabine:  12 patients achieved partial response (PR).  25 patients achieved stable disease for a median of 6 months (Mom et al., 2009). | Completed Phase 1 |
|  | Multiple myeloma | With/without bortezomib | 104 participants. No adverse side-effects.  No major benefit, similar to results of bortezomib alone. Response rate, progression-free survival (PFS) and duration of response rate compared to bortezomib alone (Belch et al., 2010). | Completed Phase 2 |
|  | Non-Hodgkin’s Lymphoma | Monotherapy | No drug-related hepatic or dose-limiting toxicity observed. 2 complete response (CR) and 1 PR achieved in a cohort of 40 patients with follicular lymphoma (FL). maptumumab is safe and promising for FL (Younes et al., 2010). | Completed Phase 2 |
|  | Non-small cell lung cancer (NSCLC) | Paclitaxel, carboplatin | No clinical benefit was observed with the addition of maptumumab to paclitaxel and carboplatin but, the combination was well tolerated (von Pawel et al., 2014). CTI- NCT00583830 | Completed Phase 2 |
|  | Hepatocellular carcinoma | Sorafenib | Ongoing study, no results currently available. CTI- NCT01258608 | Ongoing,  Phase 2 |
| Lexatumumab | Childhood solid tumours *(Ewing’s sarcoma, osteosarcoma, neuroblastoma, rhabdomyosarcoma)* | Monotherapy | No patients experienced complete response (CR) or PR (Merchant et al., 2012).  CTI-NCT00428272 | Terminated at Phase 1 |

**CTI**= Clinical trial identifier

**TRAIL-R2 (DR5) monoclonal antibodies (mAbs)**

| **Name** | **Cancer type tested** | **Combination/**  **monotherapy?** | **Major outcomes** | **Clinical Trial Phase** |
| --- | --- | --- | --- | --- |
| Tigatuzumab  (CS-1008) | Triple-negative breast cancer (TNBC) | Abraxane | Ongoing, no available results currently.  CTI- NCT01307891 | Phase 2,  ongoing |
|  | Solid malignancies and lymphomas | Monotherapy | 7 of 17 participants had stable disease. CS-1008 is well tolerated (Saleh et al., 2008). | Completed  Phase 1 |
|  | Liver cancer (hepatocellular carcinoma) | Sorafenib | The combination was well tolerated in adults, but study did not meet primary efficacy end-point (Cheng et al., 2015). CTI-NCT01033240 | Completed  Phase 2 |
|  | Ovarian cancer | Paclitaxel, carboplatin | Results not reported. CTI-NCT00945191 | Completed  Phase 2 |
|  | Pancreatic cancer | Gemcitabine | Results not reported. CTI-NCT00521404 | Completed  Phase 2 |
|  | Metastatic/ unresectable NSCLC | Paclitaxel, carboplatin | Increased median progression-free survival (PFS) in patients increased in patients with CS-1008 (5.4 months) versus placebo (4.3 months). Median overall survival (OS) was 8.4 months for CS-1008 versus 9.0 months for placebo. 24.5% of CS-1008 had PR, 22.9% placebo had PR. No CR was achieved.  The addition of CS-1008 did not improved overall efficacy of therapy (Reck et al., 2013).  CTI-NCT00991796 | Completed  Phase 2 |
|  | Metastatic colorectal cancer (CRC). | Irinotecan | Results not reported. CTI- NCT00969033 | Terminated  Phase 2 |
|  |  | FOLFIRI | Results not reported. CTI- NCT01124630 | Completed  Phase 1 |
| Conatumumab (AMG-655) | Metastatic CRC | Panitumumab | 52 participants (includes 19 WT-KRAS, 25 mutant KRAS, 8 unknown KRAS).  No dose-limiting toxicities (DLT) observed.  No objective response achieved.  PFS is WT-KRAS was 10 weeks and 7.1 weeks in mutant KRAS.  Overall survival was 7.3 months in WT-KRAS, 4.4 months in mutant KRAS.  Disease control was 8/19 participants for WT-KRAS and 4/25 for mutant KRAS.  CTI- NCT00630786 | Completed  Phase 1b/2 |
|  |  | FOLFIRI  AMG-479 (ganitumab) | Increased in the objective response rate (ORR) of conatumumab + FOLFIRI (13.7%) versus placebo (2%). ORR of ganitumab + FOLFIRI was 7.8% (Cohn et al., 2012). | Completed  Phase 2. |
|  |  | mFOLFOX6, bevacizumab | Combination was well tolerated.  Phase 2: combination did not benefit the PFS in comparison to placebo (Fuchs et al., 2013). CTI- NCT00625651 | Completed  Phase 1b/2 |
|  | Sarcoma (soft tissue) | Doxorubicin | Median PFS in phase 2 was 5.4 and 6.4 months in the conatumumab-doxorubicin and placebo-doxorubicin respectively.  The addition was safe, but did not improve the efficacy of the treatment (Demetri et al., 2012) | Completed  Phase 1/2 |
|  | Lymphoma | Vorinostat, bortezomib | Results not reported. CTI-NCT00791011 | Completed  Phase 1 |
|  | Advanced NSCLC | Paclitaxel, carboplatin (PC) | No benefit to the addition of AMG-655.  Median PFS was 5.4 months in arm 1 (PC+AMG-655 3mg/kg). 4.8 months in arm 2 (PC+AMG-655 15mg/kg) and 5.5 months in the placebo (Paz-Ares et al., 2013).  CTI- NCT00534027 | Completed  Phase 1b/2 |
|  | Pancreatic cancer | AMG-479 (ganitumab), gemcitabine | 6-month survival rates:  Gemcitabine +AMG-479 is 57%,  Gemcitabine + AMG-599 is 59%,  Placebo is 50%.  Improved 6-month survival (Kindler et al., 2012) | Completed  Phase 2 |
|  | Advanced refractory tumours | Ganitumab | No dose-limiting toxicities (DLT) in phase 1. In phase 2, 36% of patients achieved a stable disease, but no objective responses were observed (Tabernero et al., 2015)  CTI- NCT00819169 | Terminated  Phase 1/2 |
|  | Ovarian cancer | Birinapant | Results not reported. CTI- NCT01940172 | Completed  Phase 1 |
|  | Advanced solid tumours, carcinoid, colorectal cancer  locally advanced  lymphoma  metastatic cancer, NSCLC  sarcoma, solid tumours | FOLFOX6, ganitumab, bevacizumab | Results not reported. CTI- NCT01327612 | Phase 2,  Ongoing |
|  | Solid tumours | Ganitumab | No results reported. Participants rolled over to NCT01327612.  CTI- NCT00819169 | Terminated at Phase 1b/2 |
| Drozitumab (PRO95780) | Metastatic CRC | Bevacizumab, FOLFOX | Results not reported. CTI- NCT00851136 | Completed  Phase 1 |
|  |  | Bevacizumab, cetuximab, FOLFIRI, irinotecan | Of the 20 participants, there were 3 PR, 13 stable disease, 4 progressive disease. Treatment was well tolerated (Baron et al., 2011) | Completed  Phase 1 |
|  | Non-Hodgkin’s Lymphoma | Rituximab | Of the 40 participants, 20 achieved an overall response, 2 achieved complete response or unreported complete response and 18 achieved a PR (Wittebol et al., 2010). | Completed  Phase 2 |
|  | NSCLC | Bevacizumab, carboplatin, paclitaxel | Results not reported. CTI- NCT00480831 | Completed  Phase 2 |
|  | Chondrosarcoma | Monotherapy | Results not reported. CTI- NCT00543712 | Terminated at Phase 2 |

**Recombinant soluble TRAIL variants**

| **Name** | **Cancer type tested** | **Combination/**  **monotherapy?** | **Major outcomes** | **Clinical Trial Phase** |
| --- | --- | --- | --- | --- |
| Dulanermin  (AMG-951) | NSCLC | Bevacizumab, carboplatin, paclitaxel | Paclitaxel+carboplatin (PC), ORR = 39%  Dulanermin+ PC, ORR = 38%  PC + bevacizumab (PCB) =50%  PCB + dulanermin (8mg/kg) = 40%  PCB +dulanermin (20mg/kg) = 40%  Addition of dulanermin did not improve patient outcome (Soria et al., 2011).  CTI- NCT00508625 | Completed  Phase 2 |
|  | CRC | FOLFOX regimen, bevacizumab | Results not reported. CTI- NCT00873756 | Completed  Phase 1 |
|  |  | FOLFIRI regimen,  bevacizumab,  cetuximab,  irinotecan | Results not reported. CTI- NCT00671372 | Completed  Phase 1 |
|  | NHL | Rituximab | **Rituximab Alone (22 participants):** 14 total objective response. PFS is 29.9 months  **Combination (25 participants):** 16 Objective response. PFS is 17.9 months.  **Dulanermin (11 participants):** 1 objective response. PFS is 6.9 months (Cheah et al., 2015). CTI- NCT00400764 | Terminated at  Phase 2 |

**Examples of bispecifc Antibodies (BsAbs) for TRAIL**

| **Name** | **Cancer type tested** | **Combination/**  **monotherapy?** | **Major outcomes** | **Clinical Trial Phase** |
| --- | --- | --- | --- | --- |
| MCSPxDR5 | Melanoma | Monotherapy | Binds with high selectivity to cells expressing MCSP (present of 90% of melanomas), exerting DR5-mediated apoptosis/cytotoxicity.  Further, antibody has the ability to induce antibody dependent NK-cell mediated ADCC (He et al., 2016) | Pre-clinical |
| RG7386 | Solid tumours | Monotherapy or in combination with either irinotecan or doxorubicin | Antibody is comprised of agonistic DR5 binder and FAP targeting moiety. Fibroblast activation protein (FAP) is a marker for activated fibroblasts, expressed in cancer associated fibroblasts of various epithelial tumours. Antibody induces FAP dependent DR5 hyper-clustering and apoptosis induction in DR5 positive tumour cells.  Substantial tumour regression in patient-derived xenograft models (Brünker et al., 2016). Recruitment ongoing for phase 1 clinical trial in solid tumours (NCT02558140). | Advancing to phase 1 clinical trials |
| TRAIL-R2/LTβR | Breast cancer  Colon cancer | Monotherapy | Engineered from TRAIL-R2 (DR5) linked at the C-terminus to an scFv specific for lymphotoxin-beta receptor (LTβR).  Tumour cell growth was inhibited in MDA-MB-231 (breast cancer), Me180 (cervical cancer) and WiDr (colorectal adenocarcinoma).  Furthermore, in a MDA-MB-231-derieved xenograft model demonstrated significant tumour inhibition, whereas the N-terminal bsAbs did not inhibit tumour growth in the model. Furthermore, tumour growth was inhibited (over 60%) in a WiDr xenograft upon treatment with both N- and C-terminal bsAbs (Michaelson et al., 2009). | Pre-clinical |

**TRAIL fusion proteins**

| **Name** | **Cancer type tested** | **Combination/**  **monotherapy?** | **Major outcomes** | **Clinical Trial Phase** |
| --- | --- | --- | --- | --- |
| K12:TRAIL or  anti-CD3:TRAIL | Tested on various cell lines (ovarian carcinoma, colon carcinoma, melanoma, hepatocellular carcinoma) | Monotherapy | Anti-CD3:TRAIL and K12:TRAIL once added to T cells will bind to CD3 and CD7 respectively, resulting in increased surface TRAIL. In turn, these fusion protein increase T cell tumourcidal activity towards cancer cells. Furthermore, in murine xenograft models (xenografted colorectal carcinoma cell line HCT-116), tumour growth was significantly disrupted, survival-time increased 6-fold (de Bruyn et al., 2011). | Pre-clinical |
| scFvCD33:sTRAIL | leukaemia (acute myeloid (AML)/ chronic myeloid (CML)) | Gleevec or  Valproic acid, 17-AAG and mitoxantrone | Exhibited potent anti-tumour activity towards CD33+ CML cells when treatment was combined gleevec. Furthermore, in *ex vivo* patient derived CD33+AML tumour cells caused potent apoptosis in combination with valproic acid, 17-AGG and mitoxantrone (ten Cate et al., 2009). | Pre-clinical |
| scFvCD7:sTRAIL | T-cell malignanies | Vincristin, UCN-01 and cycloheximide | CD7 is expressed on most T-cell malignancies, including approximately 10% of AMLs. scFvCD7:sTRAIL exhibited enhanced toxicity towards CD7^+^ leukemic cells. More potent than the immunotoxin scFvCD7:ETA (Bremer et al., 2005) | Pre-clinical |

**Fas ligand (FasL) fusion proteins**

| **Name** | **Cancer type tested** | **Combination/**  **monotherapy?** | **Major outcomes** | **Clinical Trial Phase** |
| --- | --- | --- | --- | --- |
| CTLA4:FasL or CD40:FasL | Malignant lymphatic cells | Monotherapy | CTLA-4·FasL triggers apoptosis of cells expressing CD95 and activates caspases-3, -8, and -9. Only B7-expressing B cells responded to CTLA-4·FasL.  CD40·FasL killed only the T cells that express high levels of CD40L in conjunction with CD95 (Orbach et al., 2010). | Pre-clinical |
| MegaFasL (APO010) | Solid tumours | Monotherapy | FasL fusion protein hexameric protein consisting of 2 FasL extracellular domain trimers and the collagen domain of adiponectin ACRP30.  Results not reported. CTI- NCT00437736 | Completed Phase 1 |
| scFvCD7:sFasL | Leukaemia | Bortezomib,  L-744832. | Treatment of T-ALL, AML and PTCL with scFvCD7:sFasL induced apoptosis (Bremer et al., 2006) | Pre-clincial |
| scFvRit:sFasL | B-cell malignancies | Ritumximab (mAb) | ScFvRit:sFasL induced CD20-restricted apoptosis in malignant B-cell lines (10 of 11) and primary patient-derived malignant B cells (two non–Hodgkin lymphomas six B cell chronic lymphocytic leukemia) (Bremer et al., 2008) | Pre-clinical |

**Fas Monoclonal Antibodies (mAbs)**

| **Name** | **Cancer type tested** | **Combination/**  **monotherapy?** | **Major outcomes** | **Clinical Trial Phase** |
| --- | --- | --- | --- | --- |
| JO2 | - | N/A | Can kill thymocytes but, treatment caused fulminant hepatitis, haemorrhage or death in mice (Nishimura et al., 1997). | N/A |
| RK-8 | - | N/A | Effectively induced cell death in thymocytes, did not induce cell death in hepatocytes (Nishimura et al., 1997). | N/A |

**Examples of TNF-treatments via isolated limb perfusion (ILP).**

| **Name** | **Cancer type tested** | **Combination/**  **monotherapy?** | **Major outcomes** | **Clinical Trial Phase** |
| --- | --- | --- | --- | --- |
| L19-TNF | Melanoma | Melphalan  via mild hyperthermia ILP | L19-TNF is a immunocytokine composed of the human L19 antibody binding to extra domain B (ED-B) of fibronectin of newly formed blood vessels, and of human TNF.  7 and 10 patients received 325 µg and 650 µg of L19-TNF, respectively. CR in 5/10 patients with 650 µg (durable for 12 months in 4/5) (Papadia et al., 2013). CTI- NCT01213732 | Completed  Phase I |
| - | Soft tissue saracoma (STS) | Melphalan | Tumour necrosis factor-α (TNF) and melphalan based isolated limb perfusion (TM-ILP) were performed in 275 patients with extremity STS over a 20 years period. Reduction of dosage and the experience gained in ILP led to less local toxicity and shorter hospital stay. It remains an effective treatment strategy for STS (Deroose et al., 2015) | - |

Baron, A.D., O' Bryant, C.L., Choi, Y., Ashkenazi, A., Royer-Joo, S., and Portera, C.C. (2011). Phase Ib study of drozitumab combined with cetuximab (CET) plus irinotecan (IRI) or with FOLFIRI {+/-} bevacizumab (BV) in previously treated patients (Pts) with metastatic colorectal cancer (mCRC). *J Clin Oncol (Meeting Abstracts)* 29**,** 3581.

Belch, A., Sharma, A., Spencer, A., Tarantolo, S., Bahlis, N.J., Doval, D., Gallant, G., Kumm, E., Klein, J., and Chanan-Khan, A.A. (2010). A Multicenter Randomized Phase II Trial of Mapatumumab, a TRAIL-R1 Agonist Monoclonal Antibody, In Combination with Bortezomib In Patients with Relapsed/Refractory Multiple Myeloma (MM). *Blood* 116**,** 5031.

Bremer, E., Cate, B.T., Samplonius, D.F., De Leij, L.F.M.H., and Helfrich, W. (2006). CD7-restricted activation of Fas-mediated apoptosis: a novel therapeutic approach for acute T-cell leukemia. *Blood* 107**,** 2863-2870.

Bremer, E., Samplonius, D.F., Peipp, M., Van Genne, L., Kroesen, B.-J., Fey, G.H., Gramatzki, M., De Leij, L.F.M.H., and Helfrich, W. (2005). Target Cell–Restricted Apoptosis Induction of Acute Leukemic T Cells by a Recombinant Tumor Necrosis Factor–Related Apoptosis-Inducing Ligand Fusion Protein with Specificity for Human CD7. *Cancer Research* 65**,** 3380-3388.

Bremer, E., Ten Cate, B., Samplonius, D.F., Mueller, N., Wajant, H., Stel, A.J., Chamuleau, M., Van De Loosdrecht, A.A., Stieglmaier, J., Fey, G.H., and Helfrich, W. (2008). Superior Activity of Fusion Protein scFvRit:sFasL over Cotreatment with Rituximab and Fas Agonists. *Cancer Research* 68**,** 597-604.

Brünker, P., Wartha, K., Friess, T., Grau-Richards, S., Waldhauer, I., Koller, C.F., Weiser, B., Majety, M., Runza, V., Niu, H., Packman, K., Feng, N., Daouti, S., Hosse, R.J., Mössner, E., Weber, T.G., Herting, F., Scheuer, W., Sade, H., Shao, C., Liu, B., Wang, P., Xu, G., Vega-Harring, S., Klein, C., Bosslet, K., and Umaña, P. (2016). RG7386, a Novel Tetravalent FAP-DR5 Antibody, Effectively Triggers FAP-Dependent, Avidity-Driven DR5 Hyperclustering and Tumor Cell Apoptosis. *Molecular Cancer Therapeutics* 15**,** 946-957.

Cheah, C.Y., Belada, D., Fanale, M.A., Janikova, A., Czucman, M.S., Flinn, I.W., Kapp, A.V., Ashkenazi, A., Kelley, S., Bray, G.L., Holden, S., and Seymour, J.F. (2015). Dulanermin with rituximab in patients with relapsed indolent B-cell lymphoma: an open-label phase 1b/2 randomised study. *The Lancet Haematology* 2**,** e166-e174.

Cheng, A.-L., Kang, Y.-K., He, A.R., Lim, H.Y., Ryoo, B.-Y., Hung, C.-H., Sheen, I.S., Izumi, N., Austin, T., Wang, Q., Greenberg, J., Shiratori, S., Beckman, R.A., and Kudo, M. (2015). Safety and efficacy of tigatuzumab plus sorafenib as first-line therapy in subjects with advanced hepatocellular carcinoma: A phase 2 randomized study. *Journal of Hepatology* 63**,** 896-904.

Cohn, A.L., Tabernero, J., Maurel, J., Nowara, E., Dubey, S., Baker, N., Hei, Y.J., Galimi, F., and Choo, S. (2012). Conatumumab (CON) plus FOLFIRI (F) or ganitumab (GAN) plus F for second-line treatment of mutant (MT) KRAS metastatic colorectal cancer (mCRC). *J Clin Oncol (Meeting Abstracts)* 30**,** 534.

De Bruyn, M., Wei, Y., Wiersma, V.R., Samplonius, D.F., Klip, H.G., Van Der Zee, A.G., Yang, B., Helfrich, W., and Bremer, E. (2011). Cell surface delivery of TRAIL strongly augments the tumoricidal activity of T cells. *Clin Cancer Res* 17**,** 5626-5637.

Demetri, G.D., Le Cesne, A., Chawla, S.P., Brodowicz, T., Maki, R.G., Bach, B.A., Smethurst, D.P., Bray, S., Hei, Y.J., and Blay, J.Y. (2012). First-line treatment of metastatic or locally advanced unresectable soft tissue sarcomas with conatumumab in combination with doxorubicin or doxorubicin alone: a phase I/II open-label and double-blind study. *Eur J Cancer* 48**,** 547-563.

Deroose, J.P., Grünhagen, D.J., De Wilt, J.H.W., Eggermont, A.M.M., and Verhoef, C. (2015). Treatment modifications in tumour necrosis factor-α (TNF)-based isolated limb perfusion in patients with advanced extremity soft tissue sarcomas. *European Journal of Cancer* 51**,** 367-373.

Fuchs, C.S., Fakih, M., Schwartzberg, L., Cohn, A.L., Yee, L., Dreisbach, L., Kozloff, M.F., Hei, Y.-J., Galimi, F., Pan, Y., Haddad, V., Hsu, C.-P., Sabin, A., and Saltz, L. (2013). TRAIL receptor agonist conatumumab with modified FOLFOX6 plus bevacizumab for first-line treatment of metastatic colorectal cancer. *Cancer* 119**,** 4290-4298.

He, Y., Hendriks, D., Van Ginkel, R., Samplonius, D., Bremer, E., and Helfrich, W. (2016). Melanoma-Directed Activation of Apoptosis Using a Bispecific Antibody Directed at MCSP and TRAIL Receptor-2/Death Receptor-5. *Journal of Investigative Dermatology* 136**,** 541-544.

Kindler, H.L., Richards, D.A., Garbo, L.E., Garon, E.B., Stephenson, J.J., Rocha-Lima, C.M., Safran, H., Chan, D., Kocs, D.M., Galimi, F., Mcgreivy, J., Bray, S.L., Hei, Y., Feigal, E.G., Loh, E., and Fuchs, C.S. (2012). A randomized, placebo-controlled phase 2 study of ganitumab (AMG 479) or conatumumab (AMG 655) in combination with gemcitabine in patients with metastatic pancreatic cancer. *Annals of Oncology* 23**,** 2834-2842.

Merchant, M.S., Geller, J.I., Baird, K., Chou, A.J., Galli, S., Charles, A., Amaoko, M., Rhee, E.H., Price, A., Wexler, L.H., Meyers, P.A., Widemann, B.C., Tsokos, M., and Mackall, C.L. (2012). Phase I trial and pharmacokinetic study of lexatumumab in pediatric patients with solid tumors. *J Clin Oncol* 30**,** 4141-4147.

Michaelson, J.S., Demarest, S.J., Miller, B., Amatucci, A., Snyder, W.B., Wu, X., Huang, F., Phan, S., Gao, S., Doern, A., Farrington, G.K., Lugovskoy, A., Joseph, I., Bailly, V., Wang, X., Garber, E., Browning, J., and Glaser, S.M. (2009). Anti-tumor activity of stability-engineered IgG-like bispecific antibodies targeting TRAIL-R2 and LTβR. *mAbs* 1**,** 128-141.

Mom, C.H., Verweij, J., Oldenhuis, C.N.a.M., Gietema, J.A., Fox, N.L., Miceli, R., Eskens, F.a.L.M., Loos, W.J., De Vries, E.G.E., and Sleijfer, S. (2009). Mapatumumab, a Fully Human Agonistic Monoclonal Antibody That Targets TRAIL-R1, in Combination with Gemcitabine and Cisplatin: a Phase I Study. *Clinical Cancer Research* 15**,** 5584-5590.

Nishimura, Y., Hirabayashi, Y., Matsuzaki, Y., Musette, P., Ishii, A., Nakauchi, H., Inoue, T., and Yonehara, S. (1997). In vivo analysis of Fas antigen-mediated apoptosis: effects of agonistic anti-mouse Fas mAb on thymus, spleen and liver. *International Immunology* 9**,** 307-316.

Orbach, A., Rachmilewitz, J., Shani, N., Isenberg, Y., Parnas, M., Huang, J.-H., Tykocinski, M.L., and Dranitzki-Elhalel, M. (2010). CD40·FasL and CTLA-4·FasL Fusion Proteins Induce Apoptosis in Malignant Cell Lines by Dual Signaling. *The American Journal of Pathology* 177**,** 3159-3168.

Papadia, F., Basso, V., Patuzzo, R., Maurichi, A., Di Florio, A., Zardi, L., Ventura, E., González-Iglesias, R., Lovato, V., Giovannoni, L., Tasciotti, A., Neri, D., Santinami, M., Menssen, H.D., and De Cian, F. (2013). Isolated limb perfusion with the tumor-targeting human monoclonal antibody–cytokine fusion protein L19-TNF plus melphalan and mild hyperthermia in patients with locally advanced extremity melanoma. *Journal of Surgical Oncology* 107**,** 173-179.

Paz-Ares, L., Bálint, B., De Boer, R.H., Van Meerbeeck, J.P., Wierzbicki, R., De Souza, P., Galimi, F., Haddad, V., Sabin, T., Hei, Y.-J., Pan, Y., Cottrell, S., Hsu, C.-P., and Ramlau, R. (2013). A Randomized Phase 2 Study of Paclitaxel and Carboplatin with or without Conatumumab for First-Line Treatment of Advanced Non–Small-Cell Lung Cancer. *Journal of Thoracic Oncology* 8**,** 329-337.

Reck, M., Krzakowski, M., Chmielowska, E., Sebastian, M., Hadler, D., Fox, T., Wang, Q., Greenberg, J., Beckman, R.A., and Von Pawel, J. (2013). A randomized, double-blind, placebo-controlled phase 2 study of tigatuzumab (CS-1008) in combination with carboplatin/paclitaxel in patients with chemotherapy-naïve metastatic/unresectable non-small cell lung cancer. *Lung Cancer* 82**,** 441-448.

Saleh, M.N., Percent, I., Wood, T.E., Posey, J., Iii, Shah, J., Carlisle, R., Wojtowicz-Praga, S., and Forero-Torres, A. (2008). A phase I study of CS-1008 (humanized monoclonal antibody targeting death receptor 5 or DR5), administered weekly to patients with advanced solid tumors or lymphomas. *J Clin Oncol (Meeting Abstracts)* 26**,** 3537.

Soria, J.-C., Márk, Z., Zatloukal, P., Szima, B., Albert, I., Juhász, E., Pujol, J.-L., Kozielski, J., Baker, N., Smethurst, D., Hei, Y.-J., Ashkenazi, A., Stern, H., Amler, L., Pan, Y., and Blackhall, F. (2011). Randomized Phase II Study of Dulanermin in Combination With Paclitaxel, Carboplatin, and Bevacizumab in Advanced Non–Small-Cell Lung Cancer. *Journal of Clinical Oncology* 29**,** 4442-4451.

Tabernero, J., Chawla, S.P., Kindler, H., Reckamp, K., Chiorean, E.G., Azad, N.S., Lockhart, A.C., Hsu, C.-P., Baker, N.F., Galimi, F., Beltran, P., and Baselga, J. (2015). Anticancer activity of the type I insulin-like growth factor receptor antagonist, ganitumab, in combination with the death receptor 5 agonist, conatumumab. *Targeted oncology* 10**,** 65-76.

Ten Cate, B., Bremer, E., De Bruyn, M., Bijma, T., Samplonius, D., Schwemmlein, M., Huls, G., Fey, G., and Helfrich, W. (2009). A novel AML-selective TRAIL fusion protein that is superior to Gemtuzumab Ozogamicin in terms of in vitro selectivity, activity and stability. *Leukemia* 23**,** 1389-1397.

Von Pawel, J., Harvey, J.H., Spigel, D.R., Dediu, M., Reck, M., Cebotaru, C.L., Humphreys, R.C., Gribbin, M.J., Fox, N.L., and Camidge, D.R. (2014). Phase II trial of mapatumumab, a fully human agonist monoclonal antibody to tumor necrosis factor-related apoptosis-inducing ligand receptor 1 (TRAIL-R1), in combination with paclitaxel and carboplatin in patients with advanced non-small-cell lung cancer. *Clin Lung Cancer* 15**,** 188-196.e182.

Wittebol, S., Ferrant, A., Wickham, N.W., Fehrenbacher, L., Durbin-Johnson, B., and Bray, G.L. (2010). Phase II study of PRO95780 plus rituximab in patients with relapsed follicular non-Hodgkin's lymphoma (NHL). *J Clin Oncol (Meeting Abstracts)* 28**,** e18511.

Younes, A., Vose, J.M., Zelenetz, A.D., Smith, M.R., Burris, H.A., Ansell, S.M., Klein, J., Halpern, W., Miceli, R., Kumm, E., Fox, N.L., and Czuczman, M.S. (2010). A Phase 1b/2 trial of mapatumumab in patients with relapsed/refractory non-Hodgkin's lymphoma. *British Journal of Cancer* 103**,** 1783-1787.
